# Species Composition, Temporal Abundance and Distribution of Insect Captures Inside and Outside Commercial Peanut Shelling Facilities

**DOI:** 10.3390/insects11020110

**Published:** 2020-02-09

**Authors:** Lauren M. Perez, Patricia J. Moore, Mark R. Abney, Michael D. Toews

**Affiliations:** 1 Department of Entomology, University of Georgia, Tifton, GA 31793, USA; l.perez@uga.edu (L.M.P.); mrabney@uga.edu (M.R.A.); 2 Department of Entomology, University of Georgia, Athens, GA 30601, USA; pjmoore@uga.edu

**Keywords:** Lasioderma serricorne, Tribolium castaneum, Tribolium confusum, Cadra cautella, Plodia interpunctella, monitoring, food processing, trapping, sanitation

## Abstract

Insect populations were studied within two commercial peanut shelling facilities located in the southeastern United States. Commercially available pheromone/kairomone-baited dome traps and pheromone-baited flight traps were deployed throughout processing and shipping portions of the shelling plants and serviced weekly over one year. *Lasioderma serricorne, Tribolium castaneum, Typhaea stercorea, Carpophilus* spp., *Plodia interpunctella* and *Cadra cautella* were the most common captures across locations. *Lasioderma serricorne* made up 87% and 88% of all captures in dome traps in plants one and two, respectively. While *L. serricorne* was not captured during the winter months in flight traps, it was captured with near 100% frequency in dome traps, suggesting that populations persisted throughout the year inside the facilities. *Tribolium castaneum* populations were active year round. Across insect species and trap type, temperature was a significant covariate for explaining variation in insect counts. After accounting for the effect of temperature, there were always more insects captured in the processing portions of the facilities compared to the shipping areas. A negative linear relationship was observed between captures of *L. serricorne* and *T. castaneum* and trap distance from in-shell peanuts entering the shelling facilities. Conversely, fungivores were more evenly distributed throughout all parts of the shelling plants. These data suggest that management efforts should be focused where in-shell peanuts enter to reduce breeding and harborage sites for grain feeding insects.

## 1. Introduction

Long-term farmers’ stock peanut storage and peanut shelling facilities provide unique environments that are favorable to many stored product insect species. General sanitation in these environments is often poor, because peanut pods develop underground and must be dug from the soil at harvest. Further, farmers’ stock peanuts are prone to breakage when moved. Spilled debris, including shells and seeds, on warehouse floors or under shelling machinery can provide enough sustenance to support stored product pests, even when facilities are vacant [1]. Stored product pests have a unique ability to exploit collections of dust and food particles, environments that are very common among food processing facilities [2]. For example, stored product moths (Lepidoptera: Pyralidae) can survive on peanuts and peanut residues and will evenly distribute eggs in relation to amount of food product present [3,4]. Another common stored product pest, the red flour beetle, *Tribolium castaneum* (Herbst), can readily move between food patches and exploit patches of different sizes within food processing facilities, with 1 to 24 red flour beetles leaving large food sources every day [2]. Peanut shelling plants are not temperature or humidity controlled, and nearly all stored products pests have a high population growth rate under typical optimal conditions. Therefore, managers depend on sanitation as the backbone of their pest mitigation programs [5].

Effective integrated pest management monitoring programs require active monitoring, correct pest identification, and understanding changes in insect populations with time and temperature [6]. Insect monitoring can be conducted in two ways: direct or indirect sampling. Direct sampling, achieved by acquiring a representative commodity sample (e.g., 1 kg of shelled peanut) and enumerating total insects within, is the most accurate method to monitor for insects while commodities are in storage [7]. However, direct sampling of representative quantities is seldom practical or even possible in food processing facilities. Indirect sampling or use of sample methods that are not tied to an area of land or volume of a commodity, are more practical. Toews and Nansen [7] argue that use of pheromone-baited traps is the best way to identify and estimate insect infestations in processing, warehousing and retail environments. Insect populations within processing facilities are difficult to study due to constant movement of food sources [1]. Since insects tend to travel along cracks, crevices, and edges in search of mates or food patches [2], food lure and/or pheromone-baited traps can be deployed where direct sampling is impracticable [8]. Previous research shows that pheromone-baited traps allow for earlier detection and more precise population monitoring [8,9].

Pest management strategies are more effective when managers are fully informed of pest populations within their facility. Knowing species present and location of infestations provides necessary information for targeted intervention or need for reformed daily practices [7]. While routine inspections and visual monitoring can provide evidence of insect activity, insects are most active at night and are far more likely to encounter traps than to be directly observed at a given time [10]. Arthur et al. [11] proposed that the distribution of insect captures in a facility could indicate problematic areas. Widespread captures of insects over the entire facility may suggest that a “global intervention,” such as a fogging or fumigation treatment is necessary [5]. Other common management tactics used within peanut shelling facilities to mitigate insect populations include exclusion, sanitation, mating disruption, and insecticide applications; those applications may include fogging (aerosolized liquids), fumigation (insecticides applied as a gas), and residual (liquid) applications.

Sanitation is the backbone of stored product insect pest management programs. For example, lack of good sanitation within food processing facilities resulted in an average 16-fold decrease in fumigation efficacy, along with a 3-fold decrease in aerosol treatment efficacy [12]. Hagstrum and Flinn [13], mention that the removal of grain/legume residues through routine sanitation can reduce residual insect populations and reduce the risk of infested commodities by residential populations. Facility design plays a key role in analyzing sanitation needs, as equipment and building structure are rarely uniform across food processing [14]. Similarly, simple building repairs or alterations (e.g., sealing exterior or interior holes, securing door sweeps) could be of benefit in countering immigrating insects.

Previous studies documented insect pests found within many types of food processing facilities [5,15,16,17], but rigorous studies focused on long-term population trends within commercial peanut shelling facilities are lacking. This is likely due to the difficult nature of trapping in operational shelling facilities that constantly generate excessive dust and debris. However, excessive insect infestations have serious implications for food processing facilities and data are required to assist managers with decision support. Therefore, the goal of the current study was to identify species and seasonal population trends within commercial peanut shelling facilities located in the southeastern United States. This study was designed to address the following research questions: (1) are stored product pests present in all locations of facilities, (2) what species were captured between two trap types, (3) does temperature influence changes in capture intensity, (4) are there differences in captures between processing and shipping areas of each plant, and (5) how does trap distance from where in-shell peanuts entering the plant affect insect abundance?

## 2. Materials and Methods

### 2.1. Study Sites

This study was conducted in two commercial peanut shelling facilities located in the southeastern United States. Although the two facilities performed the same basic function, they were operated by different companies and featured vastly different building construction and equipment configurations. Plant 1 was a pre-engineered steel building erected in 1992 from steel columns, steel trusses and light gauge steel siding with an area of 1.37 million cubic feet under one roof. This facility contained two levels of shelling machinery with solid flooring between floors, except for cutouts where bucket elevators and conveyors moved product between floors. Trapping began at shelling plant 1 on 30 August 2018 and concluded on 27 August 2019. Plant 2 was a masonry building with steel trusses assembled from load bearing cinderblock walls that supported a steel roof; this facility was constructed in 1967 and contained 1.5 million cubic feet across three levels of shelling machinery separated by expanded metal grate flooring. Plant 2 was also equipped with a bulk railcar loading area that utilized overhead rollup doors that opened into the main floor of the shelling facility. Trapping at shelling plant 2 began on 30 October 2018 and concluded on 8 October 2019. Each plant was operated 24 h a day, seven days a week. One day per week, peanut sizing and shelling equipment was powered down for a few hours while accumulated shells and debris were vacuumed from under the equipment; concrete floors throughout the shelling plants were cleaned with a broom and compressed air every day.

### 2.2. Traps

Crawling insects were monitored with 24 dome traps (Storgard^®^ Dome^TM^ Quick-Change^TM^ trap design Trécé, Inc., Adair, OK, USA), baited with a multi species kairomone attractant and pheromone lure (Storgard^®^ Dome^TM^ Quick-Change^TM^ Ultra-Combi^TM^ ReBait^TM^ Kit, Trécé, Inc., Adair, OK, USA), that were deployed inside each facility. Dome traps were strategically positioned around the interior perimeter of the building and under shelling equipment when possible. The traps were secured to floors inside each facility via a Storgard^®^ Dome^TM^ trap holder (Trece Inc., Adiar, OK, USA) that was attached to the floor using 100% silicone caulk. To facilitate a strong bond between the floor and trap holder, that area of the floor was first prepared by cleaning the concrete or metal with a razor scraper followed by agitation with a stiff wire bristle brush and finally three quick applications of acetone that were rubbed dry using white terry cloth towels. On a weekly basis throughout the study, dome trap Quick Change^TM^ pheromone trap bottoms were removed and placed in plastic Petri-dishes (10 cm diameter), and a clean trap bottom was replaced on the trap. Used trap bottoms were transported to the lab for insect identification and enumeration under 20x magnification. All trap bottoms were discarded and replaced after six weeks to maintain fresh pheromone lures. In cases where excessive peanut dust filled the trap bottoms, the dust was scraped out and five drops of Storgard^®^ attractant oil (part ST/CA/3320-00, Trece Inc., Adair, OK, USA) were applied to the filter paper, lining the interior of quick-change bottoms, to maintain attractiveness of the kairomone oil during the next deployment. Although every effort was made to place traps in locations where they were difficult to kick or break loose, a few traps occasionally went missing and had to be removed from the dataset. Trap position in each facility was determined by measuring the distance to each wall using a laser distance meter.

Concurrent with the dome traps for crawling insects, flying insects were monitored inside each shelling facility through deployment of 12 Pherocon^®^ Delta III Traps (Trece Inc., Adair, OK, USA) baited with a Storgard^®^ Cap IMM+4 moth lures and Storgard^®^ Cap KB/WB beetle lures (Trece Inc., Adiar, OK, USA). Further, 6 identically baited Pherocon^®^ Delta III Traps were deployed around the exterior property line of each shelling facility to monitor the presence of exterior insect populations. Exterior traps were located at least 200 m from the shelling plants. Similar to dome traps, all flight traps were collected weekly and a fresh trap was replaced in the same spot. Pheromone lures were replaced every six weeks to maintain trap attractiveness.

At each trap-servicing interval, all insects were removed, and adults were enumerated and identified to species using available dichotomous keys for stored product insects [18,19,20]. Incidental or non-target insect captures (i.e., members of the Carabidae, Scarabaeidae or Elateridae) were identified to family following the taxonomic key of Triplehorn and Johnson [21]. All traps (dome and flight) and pheromone tray bottoms were labeled with a number and sorted based on location within or outside of the facility. Facilities were broken up into two general areas: processing and shipping (including warehouse and rail areas).

### 2.3. Temperature and Relative Humidity Monitoring

Temperature and relative humidity (RH) were monitored throughout the study inside the facilities with HOBO^®^ temp/RH loggers (model UX100-011A2, Onset Computer Corp., Bourne, MA, USA). One logger was placed in the processing portion and one in the shipping portion of each of the commercial peanut shelling facilities. Loggers were set to record temperature every thirty minutes. For presentation on the figures shown below, average daily temperature and RH are graphically displayed; for utilization in statistical tests, the average temperature for that trapping interval was used. Outdoor temperature was not recorded at either facility.

### 2.4. Statistical Analyses and Data Presentation

Sum insect captures and capture frequency (intervals with at least one capture/total trapping intervals) were summarized for each species. Commonly occurring insects captured in dome and flight traps were depicted using line graphs that illustrate mean and standard error captures per trap per week. In the unusual event that the trapping interval was not exactly 7 days, weekly capture means were adjusted by dividing the counts during the interval by the number of days that the traps were deployed and then multiplying that quotient by seven to obtain an adjusted weekly average [5]. These calculations were made using PROC MEANS [22] with adults captured as the response variable [5].

Separate analyses were conducted by facility, as there were profound differences in building design and construction materials, age of equipment inside facilities, and management practices between facilities. Prior to statistical analyses, a logarithmic transformation [X’ = log (X + 1)] was used to normalize distributions and meet assumptions for equal variance [23]. While the tables account for total numbers of insects collected and the relative frequency of those captures, formal statistical tests were performed for the four most abundant species captured. Those tests included a formal comparison of captures, by species and trap type, between the processing and shipping areas of each plant, and an assessment of how captures varied by approximate trap position as measured by linear distance from in-shell peanuts entering the facility. The comparison between shelling and processing was conducted using a repeated measures analysis of covariance, with temperature as a covariate [PROC GLIMMIX (22)]. There were two random terms in the model, trap(location) and date/subject = trap*location; a compound symmetry covariance structure fit the data better than other candidates. Distance between in-shell peanuts entering the facility and trap location was assessed by regressing insect counts on distance [PROC REG, (22)].

## 3. Results

### 3.1. Plant 1—Insect Species and Abundance

**Figure 1:**
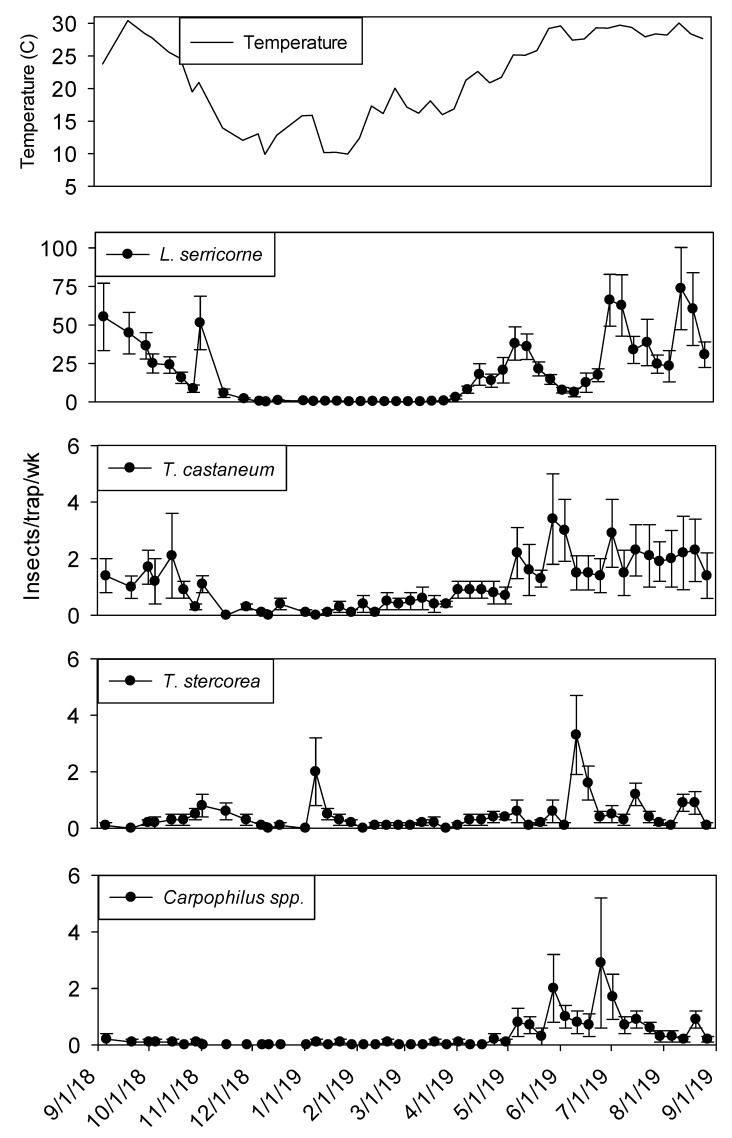
Mean temperature and mean + SEM of adult *L. serricorne*, *T. castaneum, T. stercorea* and *Carpophilus* spp. captured in dome traps located in processing portion of peanut shelling plant 1. Y-axis varies by species.

Capture of beetles and moths within dome traps was highly variable over the yearlong sampling period. Within shelling plant 1, *Lasioderma serricorne* (F.) was the most abundant species in terms of sum captures and capture frequency. The second most frequently captured beetle was *T. castaneum* followed by *Typhaea stercorea* (L.) and *Carpophilus* spp., which were captured in 90% and 67% of trapping intervals, respectively (Table 1). Approximately 16 times more *L. serricorne* were captured in dome traps compared to *T. castaneum* adults, and 38 times more than *T. stercorea* and *Carpophilus* spp. Although captures of all insect species were relatively low from November through April, there was a peak in *T. stercorea* captures in mid-January (Figure 1). While capture of *L. serricorne* and *Carpophilus* spp. were very close to zero from December through April, *T. castaneum* and *T. stercorea* were captured throughout this period.

Overall abundance of insect captures in flight traps inside shelling plant 1 was considerably less than dome traps. Flight traps located in shelling plant 1 captured similar numbers of *Cadra cautella* (Walker) and *Plodia interpunctella* (Hübner) (Table 1). Both *C. cautella* and *P. interpunctella* were found in >70% of indoor trapping intervals, while *L. serricorne* and *Trogoderma variabile* Ballion were captured in 52% and 29% of trapping intervals, respectively. All other remaining taxa found in flight traps were captured in ≤15% of trapping intervals. Flight traps located around the exterior of shelling plant 1 captured a large variety of incidental insects such as members of the Carabidae, Cicadellidae, Staphylinidae, and Scarabaeidae (Table 1). *Trogoderma variabile* was the most frequently captured species (78% of trapping intervals) followed by Staphylinidae (70% of trapping intervals). Cicadellidae, *C. cautella*, *P. interpunctella*, and Scolytidae were captured in at least 30% of trapping intervals.

Temperature in plant 1 ranged from 10 to 30° C during the study. Interior temperatures remained below 20° C from November through February, although there was an unseasonably warm temperature peak in mid-January. From June through October temperatures remained above 25° C. Maximum temperatures were observed from late May through early September.

### 3.2. Plant 1—Population Trends

**Figure 2:**
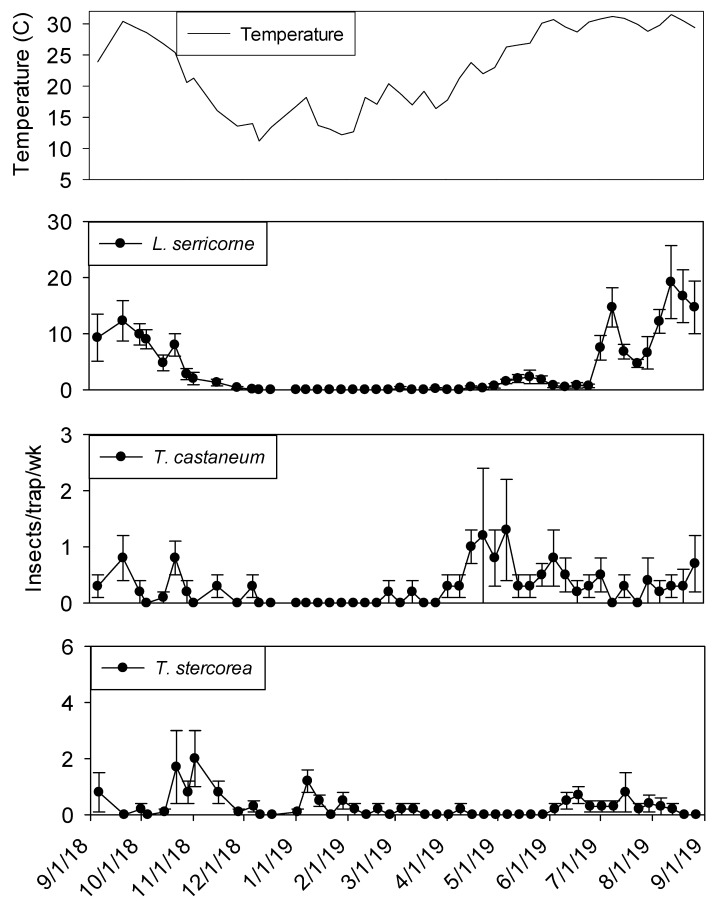
Mean temperature and mean + SEM of adult *L. serricorne*, *T. castaneum* and *T. stercorea* captured in dome traps located in shipping portion of peanut shelling plant 1. Y-axis varies by species.

In both processing and shipping portions of shelling plant 1, dome trap captures were very low during the winter months before reaching maximum levels from June through August (Figure 1 and Figure 2). Temperature was a statistically significant covariate when analyzing captures in dome traps for *L. serricorne* (F = 850.48; df = 1, 905; *p* < 0.01)*, T. castaneum* (F = 138.44; df = 1, 905; *p* < 0.01)*, T. stercorea* (F = 7.12; df =1, 905; *p* < 0.01) and *Carpophilus* spp. (F = 108.21; df = 1, 904; *p* < 0.01). There were significantly more *L. serricorne* captured in the processing area compared to shipping area (F= 17.11; df = 1, 18; *p* < 0.01). Although, *L. serricorne* was nearly absent in processing traps from December 2018 to April 2019, they were the most abundant pest found in dome traps located in the processing and shipping portions of plant 1. *Lasioderma serricorne* reached a maximum average of 75 beetles per trap per week in processing and 20 beetles per trap per week in shipping. There were three defined *L. serricorne* peaks in processing traps beginning in May 2019 and two peaks in shipping nearly two months later into the summer. Significantly more *T. castaneum* were captured in dome traps located in the processing portion of plant 1 compared to shipping (F = 4.45; df = 1, 18; *p* = 0.05). *Tribolium castaneum* was captured throughout the year in processing traps, although captures were very low from mid-November to April. In comparison, there were no differences between trap locations for *T. stercorea* (F = 0.58; df = 1, 18; *p* = 0.46) and *Carpophilus* spp. (F = 2.84; df = 1, 18; *p* = 0.11) in dome trap captures.

**Figure 3:**
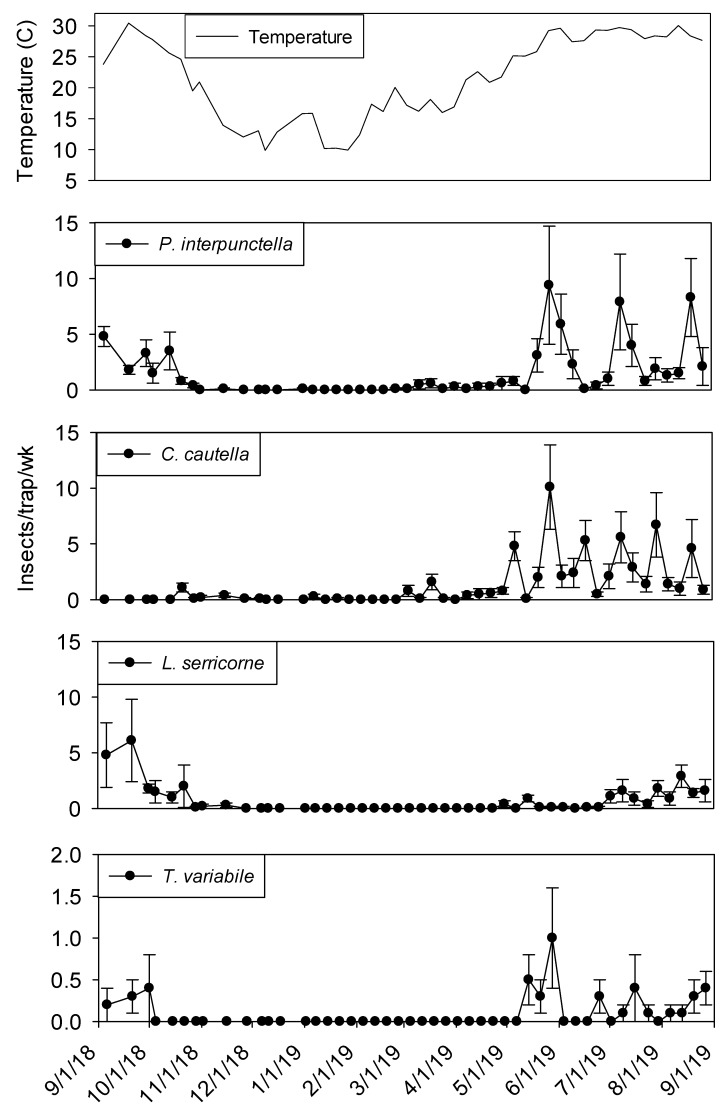
Mean temperature and mean + SEM of adult *P. interpunctella*, *C. cautella*, *L. serricorne* and *T. variabile* captured in flight traps located in processing portion of peanut shelling plant 1. Y-axis varies by species.

**Figure 4:**
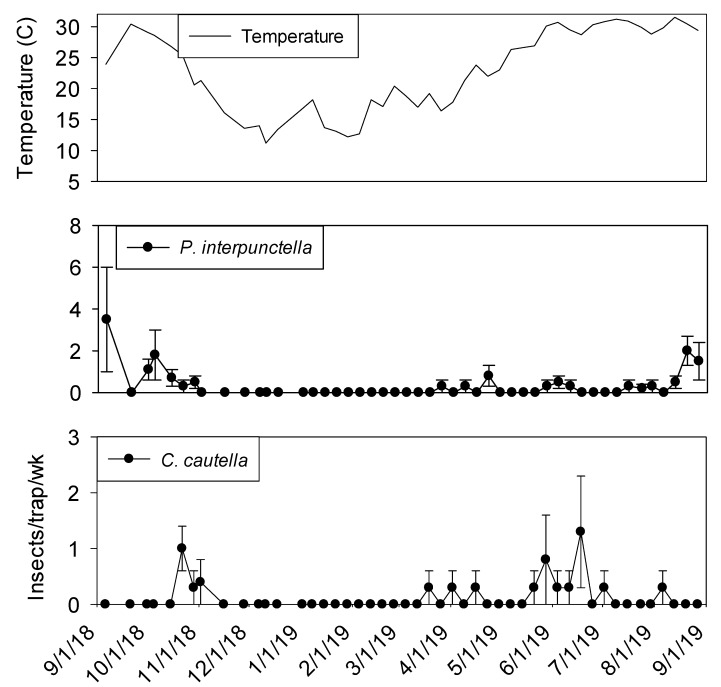
Mean temperature and mean + SEM of adult *P. interpunctella* and *C. cautella* captured in flight traps located in shipping portion of peanut shelling plant 1. Y-axis varies by species.

Temperature was a significant covariate on indoor flight trap captures for *P. interpunctella* (F = 138.90; df = 1, 559; *p* < 0.01), *C. cautella* (F = 82.67; df = 1, 559; *p* < 0.01), *L. serricorne* (F = 68.46; df = 1, 559; *p* < 0.01), and *T. variabile* (F = 24.4; df = 1, 559; *p* < 0.01). Few insects were captured in flight traps when temperatures fell below 15° C in the processing and shipping portions of plant 1 (Figure 3 and Figure 4). Flight trap captures did not resume in processing until mid-March for *P. interpunctella* and *C. cautella* and May for *L. serricorne* and *T. variabile*. Highest captures of *P. interpunctella* correspond with high peaks for *C. cautella* in June, August, and September 2019, both reaching 10 insects per trap per week in June. *Lasioderma serricorne* was captured most frequently in late fall of 2018 when temperature reached 30 °C. *Trogoderma variabile* was not captured after temperatures fell below 27° C in 2018 and returned when temperature rose above 25° C in May of 2019 (Figure 3). More captures were observed from traps in processing compared to shipping for *L. serricorne* (F = 7.80; df = 1, 10; *p* = 0.02) and *C. cautella* (F = 4.78; df = 1, 10; *p* = 0.05). There were no differences in trap capture between shipping and processing for *P. interpunctella* (F = 4.16; df = 1, 10; *p* = 0.07) or *T. variabile* (F = 2.21; df = 1, 10; *p* = 0.17).

**Figure 5:**
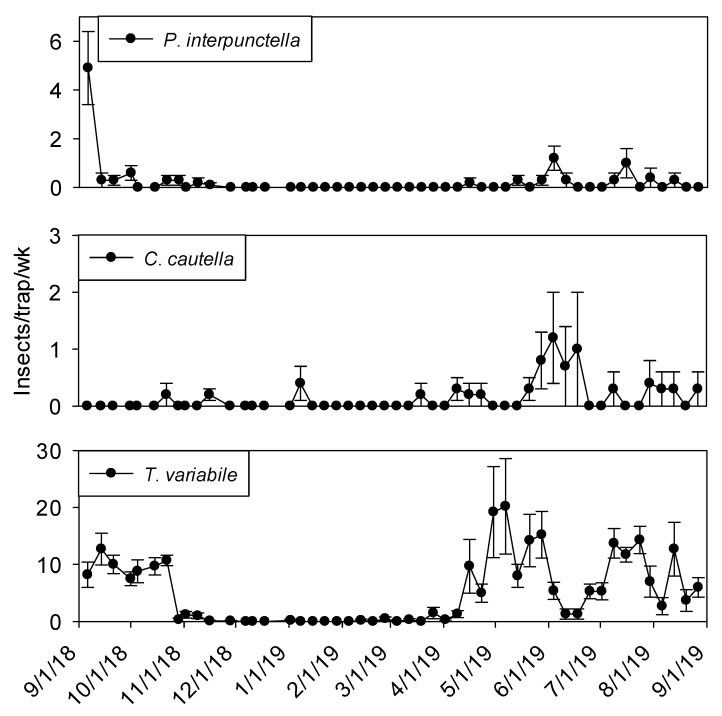
Mean + SEM of adult *P. interpunctella*, *C. cautella* and *T. variabile* captured in outdoor flight traps surrounding peanut shelling plant 1. Y-axis varies by species.

Few insects were captured in exterior flight traps during winter months (Figure 5). Fewer *P. interpunctella* were collected in September 2019 compared to September 2018. *Cadra cautella* were present at very low levels during the winter months and captures did not start increasing until mid-May. More than 10 *T. variabile* per trap per week were observed from May through June.

### 3.3. Plant 1—Relationship between Insect Abundance and Trap Distance from in-shell Farmers’ Stock Peanut Entrance

There was a strong negative linear relationship (F = 17.52; df = 1, 18; r^2^ = 0.49; *p* < 0.01) between sum counts of *L. serricorne* and dome trap distance from incoming farmers’ stock peanuts. The equation of the line was *y* = −4.20 (±1.00) *x* + 1442.78 (±211.78). There was also a strong negative linear relationship (F = 4.26; df = 1, 18; r^2^ = 0.19; *p* = 0.05) between sum counts of *T. castaneum* and dome trap distance from incoming unshelled peanut product. The equation of the line was *y* = −0.22 (±0.11) *x* + 80.1 (±22.09). Conversely, there was no trap distance relationship between sum counts of *T. stercorea* (F = 0.02; df = 1, 18; r^2^ = 0.00; *p* = 0.89), *T. variabile* (F = 1.07; df = 1, 18; r^2^ = 0.06; *p* = 0.31), and *Carpophilus* spp. (F = 3.43; df = 1, 18; r^2^ = 0.16; *p* = 0.08).

### 3.4. Plant 2—Insect Species and Abundance

Extremely high numbers of insects were captured in dome traps positioned in shelling plant 2. *Lasioderma serricorne* and *T. castaneum* were most frequently observed, both present in 100% of trapping intervals (Table 2). *Lasioderma serricorne* was the most abundant species, with 23 times more adults compared to *T. castaneum*. *Carpophilus* spp. and *Cryptolestes* spp. were the third and fourth most abundant species (1439 and 946 adults), respectively. Another *Tribolium* species, *T. confusum* Jacquelin du Val was present in 93% of trapping intervals. *Typhaea stercorea*, *A. advena*, and *Oryzaephilus mercator* (Fauvel) were captured in >64% of trapping intervals, but overall numbers of these species were very low. The remaining taxa were captured in <50% of trapping intervals.

Relatively few species were captured in indoor flight traps located in shelling plant 2. *Cadra cautella* was the most abundant and frequently captured species in indoor flight traps, found in 89% of trapping intervals (Table 2). *Lasioderma serricorne* was identified in 57% of flight trap intervals. *Plodia interpunctella*, *T. variabile*, and *Cryptolestes* spp. were captured in indoor flight traps less than 45% of all trapping intervals. Flight traps located around the exterior of shelling plant 2 captured *C. cautella* most frequently and *T. variabile* in greatest abundance (Table 2). *Plodia interpunctella* was found in 61% of trapping intervals and was the third most abundant species for outdoor flight traps. *Cryptolestes* spp. and Staphylinidae were found in similar abundances and frequencies, identified in 50% and 59% of trapping intervals, respectively. No *L. serricorne* were captured in outdoor flight traps.

### 3.5. Plant 2—Population Trends

**Figure 6:**
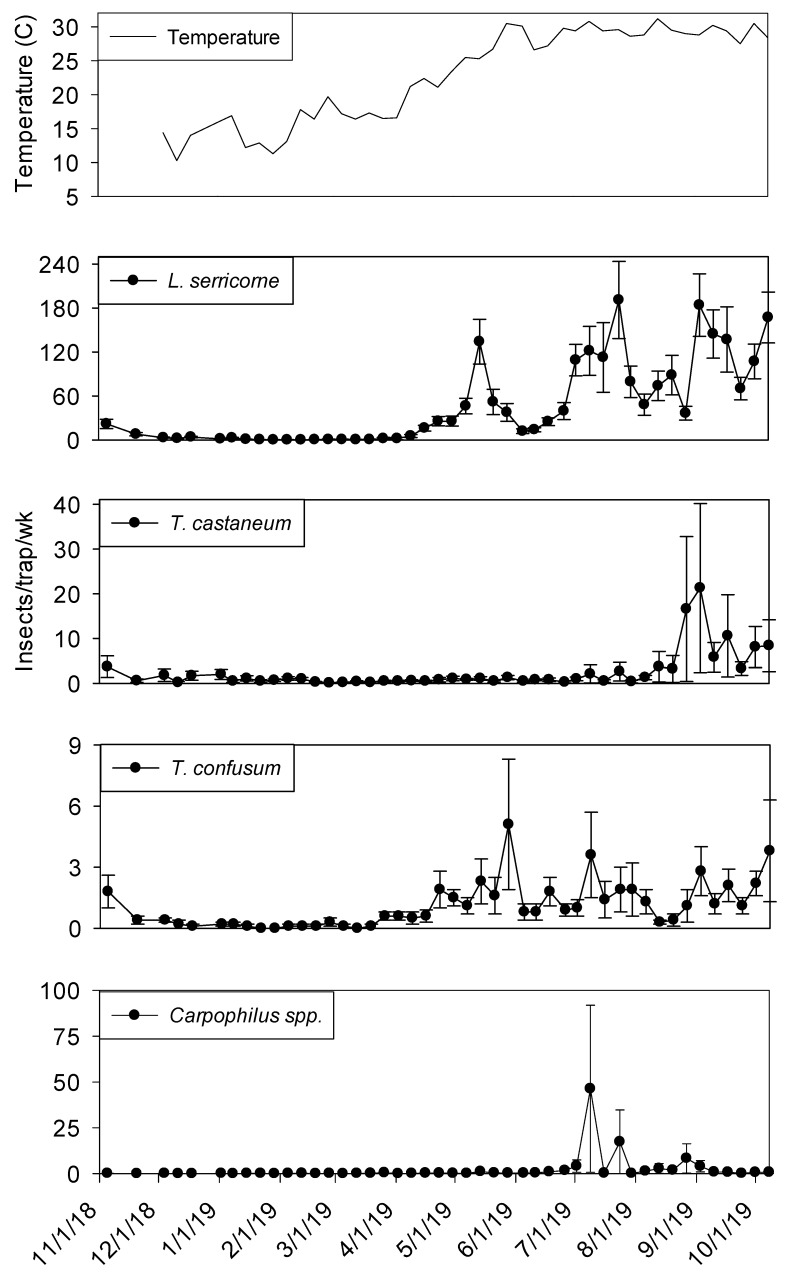
Mean temperature and mean + SEM of adult *L. serricorne*, *T. castaneum*, *T. confusum* and *Carpophilus* spp. captured in dome traps located in processing portion of peanut shelling plant 2. Y-axis varies by species.

**Figure 7:**
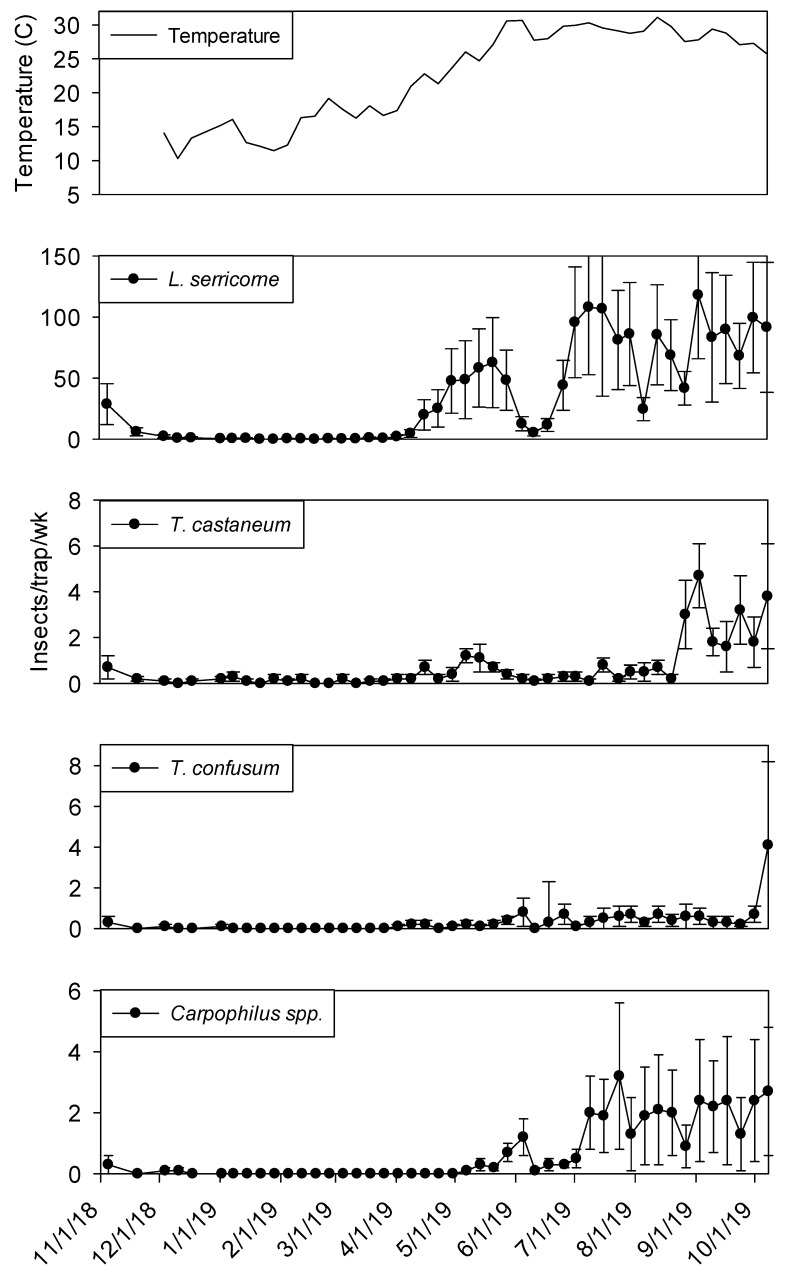
Mean temperature and mean + SEM of adult *L. serricorne*, *T. castaneum*, *T. confusum* and *Carpophilus spp* captured in dome traps located in shipping portion of peanut shelling plant 2. Y-axis varies by species.

Temperature records followed similar trends in processing and shipping portions of shelling plant 2, with lowest temperatures in late December 2018 at 10° C and highest in late May 2019 at 30° (Figure 6 and Figure 7). Temperature was a highly significant covariate on dome trap insect captures for *L. serricorne* (F = 1527.86; df = 1, 954; *p* < 0.01), *T. castaneum* (F = 43.28; df = 1, 955; *p* < 0.01), *T. confusum* (F = 146.70; df = 1, 955; *p* < 0.01) and *Carpophilus* spp. (F = 100.87; df = 1, 956; *p* < 0.01). Populations remained active in plant 2 dome traps in the processing area until mid-December 2018 for *L. serricorne*, *T. castaneum*, and *T. confusum* (Figure 6). The same trend was observed for dome traps in the shipping area for all aforementioned species with the addition of *Carpophilus* spp. (Figure 7).

Captures of *L. serricorne* were largest in the processing portion of shelling plant 2 during the months of May, July, and September 2019, reaching 120, 180, and 170 insects per trap per week, respectively. There was a large decline in *L. serricorne* captures during the months of June and August 2019 for dome traps located in processing. With large amounts of variation among traps, more *L. serricorne* were captured in the shipping portion of shelling plant 2 during the months of May, July, and September 2019, reaching 60, 100, and 120 insects per trap per week, respectively. Captures of *L. serricorne* in the shipping area declined during the month of June and again briefly in the month of August 2019. Across time, traps placed in the processing portion of the plant captured marginally more *L. serricorne* than traps placed in the shipping areas (F = 3.39; df = 1, 22; *p* = 0.08).

*Tribolium castaneum* maintained low numbers throughout this study for dome traps placed in the shipping portion of shelling plant 2 (Figure 7). Traps placed in the processing portion of shelling plant 2 reached 20 insects per trap per week (with large variation among traps) in late August 2019, declining to 10 insects per trap per week for the remainder of the study (Figure 6). Consistent populations of *T. confusum* were identified in processing traps, although at very low numbers, throughout the study for shelling plant one and two. There were significantly more *T. confusum* captured in processing compared to shipping (F = 6.81; df = 1, 22; *p* = 0.02). *Carpophilus* spp. maintained low numbers in the processing and shipping portions of shelling plant 2, with one peak reaching 50 insects per trap per week in processing with large variation among traps. There was no significant effect of trap location (processing vs. shipping) on total captures for *T. castaneum* (F = 1.50; df = 1, 22; *p* = 0.23) or *Carpophilus* spp. (F = 0.07; df = 1, 22; *p* = 0.80).

**Figure 8:**
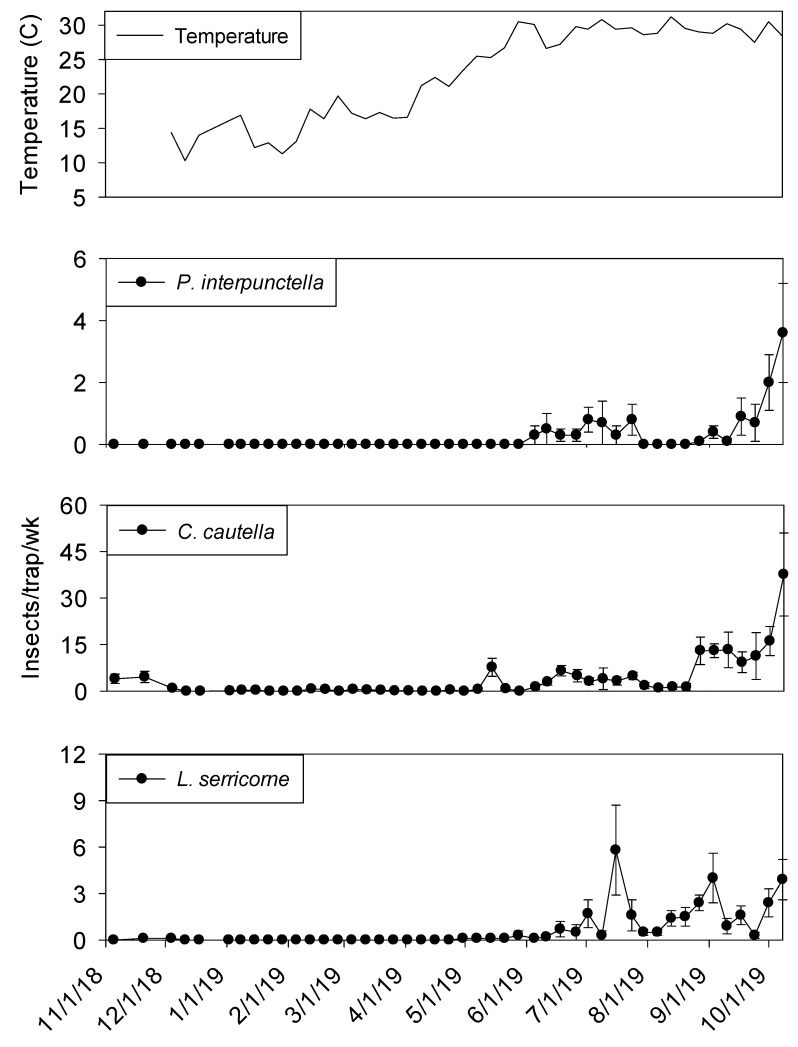
Mean temperature and mean + SEM of adult *P. interpunctella*, *C. cautella* and *L. serricorne* captured in flight traps located in processing portion of peanut shelling plant 2. Y-axis varies by species.

**Figure 9:**
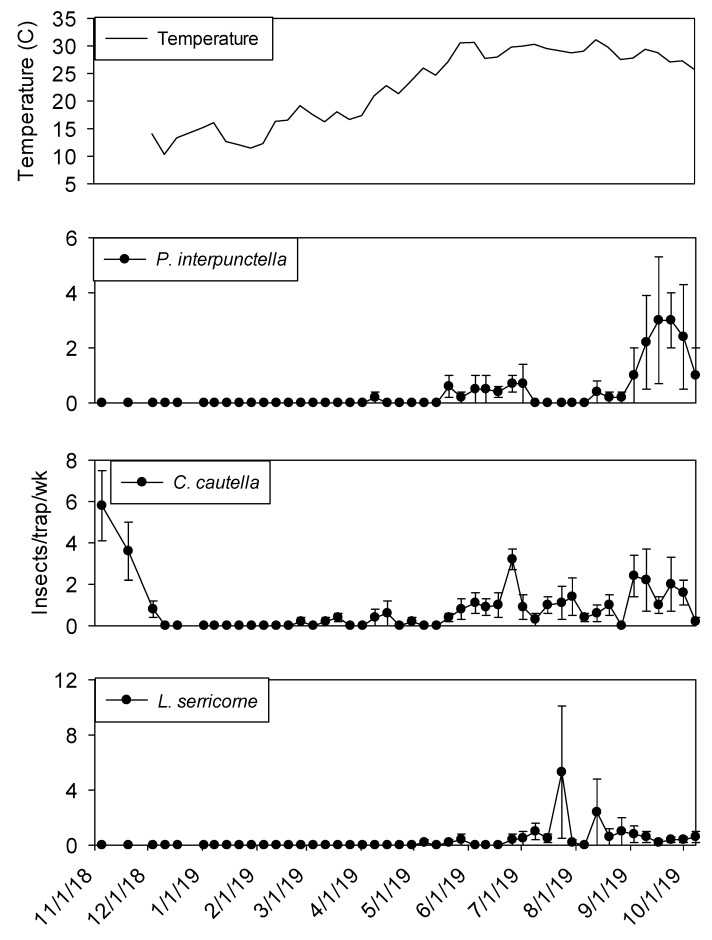
Mean temperature and mean + SEM of adult *P. interpunctella*, *C. cautella* and *L. serricorne* captured in flight traps located in shipping portion of peanut shelling plant 2. Y-axis varies by species.

Temperature was a significant covariate of indoor flight trap insect captures for *P. interpunctella* (F = 60.03; df = 1, 506; *p* < 0.01), *C. cautella* (F = 136.15; df = 1, 506; *p* < 0.01), *L. serricorne* (F = 97.94; df = 1, 506; *p* < 0.01), and *T. variabile* (F = 24.26; df = 1, 506; *p* < 0.01). *Cadra cautella* populations remained active in plant 2 processing flight traps until the beginning of December 2018, before declining during the cooler months and then returning in May. All other species were absent from traps until the beginning of June 2019 (Figure 8). In shipping, similar trends were observed for all species (Figure 9).

The three most common insects captured in processing and shipping flight traps for shelling plant two were *C. cautella*, *P. interpunctella*, and *L. serricorne* (Figure 8 and Figure 9). The most abundant pest, *C. cautella,* was the only species captured in November 2018 and the first species captured in May 2019. There were three small population increases for *C. cautella* during early summer to almost five insects per trap per week, with the largest increase beginning the first week of September and continuing through the second week of October. *Plodia interpunctella* populations were low during most of summer 2019 and began increasing at the beginning of September before reaching a peak in the second week of October 2019. *Lasioderma serricorne* was not captured in flight traps until late May 2019 in either processing or shipping. Peaks were observed in processing flight traps during July, September, and October at six, five, and five insects per trap per week, respectively. Populations of *L. serricorne* remained low in shipping flight traps throughout summer and fall months, with one increase in late July to five insects per trap per week, before decreasing to about one insect per trap per week. Significantly more *C. cautella* were captured in processing flight traps compared to shipping (F = 15.94; df = 1, 10; *p* < 0.01)*,* while no significant effect of location was observed for *P. interpunctella* (F = 0.16; df = 1, 10; *p* = 0.70) or *L. serricorne* (F = 3.23; df = 1, 10; *p* = 0.10).

**Figure 10:**
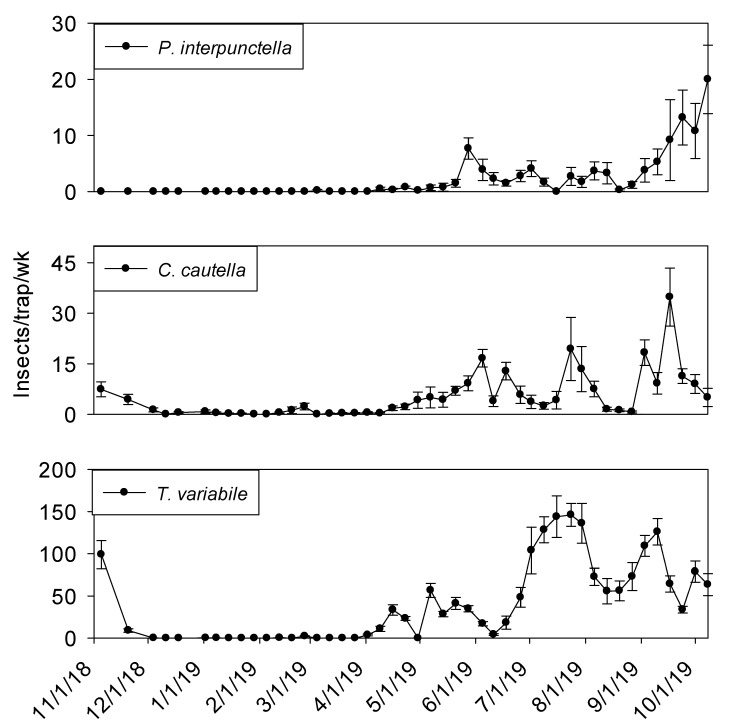
Mean + SEM of adult *P. interpunctella*, *C. cautella* and *T. variabile* captured in outdoor flight traps surrounding peanut shelling plant 2. Y-axis varies by species.

All outdoor insect captures were very low from December 2018 to April 2019, Captures initially started increasing during the second week of April (Figure 10). *Trogoderma variabile* was the most abundant species captured in outdoor flight traps with populations reaching a maximum peak in mid-July 2019 of 150 insects per trap per week. A second peak in the *T. variabile* population occurred after the first week of September 2019, reaching 125 insects per trap per week. *Cadra cautella* populations had three general increases during summer months topping out in May, July, and September at 15, 20 and 35 insects per trap per week, respectively. *Plodia interpunctella* were not captured in traps during 2018 and had the first population peak in late May 2019, reaching eight insects per trap per week. Populations were relatively low (<5 insects per trap per week) until the beginning of September 2019. Populations reached their maximum in early October.

### 3.6. Plant 2—Relationship between Insect Abundance and Trap Distance from in-shell Farmers’ Stock Peanut Entrance

There was a marginal negative linear relationship (F = 3.09; df = 1, 19; r^2^ = 0.14; *p* < 0.09) between sum counts of *L. serricorne* and dome trap distance from in-shell peanuts. The equation of the line was *y* = −6.25 (±3.55) *x* + 2713.09 (±619.44). There was a similarly marginally significant negative linear relationship (F = 3.72; df = 1, 20; r^2^ = 0.16; *p* = 0.07) between sum counts of *T. castaneum* and dome trap distance from incoming unshelled peanut product. The equation of the line was *y* = −1.05 (±0.55) *x* + 247.98 (±94.85). There was a strong negative association between capture density and trap distance for *T. confusum* (F = 6.70; df = 1, 20; r^2^ = 0.25; *p* < 0.02) and the equation of the line was *y* = -0.30 (±0.12) *x* + 84.62 (±20.32). There was no distance relationship between sum counts of *T. stercorea* (F = 0.47; df = 1, 20; r^2^ = 0.02; *p* = 0.50), *T. variabile* (F = 0.01; df = 1, 20; r^2^ = 0.01; *p* = 0.92), and *Carpophilus* spp. (F = 2.40; df = 1, 20; r^2^ = 0.11; *p* = 0.14).

## 4. Discussion

Previous authors have demonstrated that *T. castaneum* is active in both warehouse and food processing facilities year round [2,5,24]. These are similar findings to ours in that both peanut shelling facilities harbored year-round *Tribolium spp.* populations, while *L. serricorne* populations trailed off during the winter months. LaHue et al. [25] documented several of the same species in peanut warehouses as found in shelling plants in this study. Due to *T. castaneum* captures occurring year long, we hypothesize that this species is resident within the facility. Similar to Edde [26], we found that *L. serricorne* captures increased from July to December. While *T. castaneum* adults are long lived (up to 3 years), *L. serricorne* adults are very short lived [27] and a lack of reproduction combined with short lived adults likely explains capture differences between these two species in winter. Temperature was a very strong covariate of trap captures, which is consistent with other findings, as insect activity is decreased in cold climates [16,28,29]. Neither *T. stercorea* nor *Carpophilus* spp. were present during the winter months. The authors hypothesize that these species may not be resident in the facilities and could be entering through building openings or with incoming in-shell peanuts.

The most abundant species captured in this study were all grain/legume feeders. For example, *L. serricorne* and *T. castaneum* were the most abundant and most frequently captured insects in dome traps in plant 1. Similarly, *L. serricorne*, *T. castaneum*. *T. confusum* and *Cryptolestes* spp. were the most common captures in plant 2. Further, *L. serricorne*, *C. cautella and P. interpunctella* were the most common captures in flight traps across plants. The only fungus feeders that occurred in large numbers were *Carpophilus* spp. in plant 2. While fungus feeders are problematic from the perspective of live insect presence, grain/legume feeders are problematic because they could directly infest and consume commodities, including finished products [2].

These findings are in general agreement with a similar survey of insect abundance and distribution within peanut shelling plants conduced 50 years earlier [30]. Insect detection in that study utilized insects sieved from residual debris in shelling plants or insects that were incubated from fresh 453 g shelled peanut samples placed in wooden trays that were deployed around shelling plants for 3 d during late summer, fall, winter, spring and early summer. Payne and Redlinger [30] found that *C. cautella, P. interpunctella, T. castaneum, Carpophilus spp,* and *O. mercator* were the most abundant species recovered during the two year study. Further, they detected maximum insect populations in the fall and minimum populations in the winter. Interestingly, there were no *L. serricorne* or *T. variabile* insect recoveries in that study. The contrast in captures between that study and the present study is likely due to the use of pheromone attractants in traps.

We identified a negative linear relationship between the number of stored product feeding insects (*L. serricorne, T. castaneum, T. confusum, Cryptolestes* spp.) captured in dome traps and the distance from in-shell peanuts coming into the facility. Interestingly, this trend was not evident for any of the fungal feeding species, which were present at similar numbers throughout each facility. One interpretation would be that grain feeding insects are being delivered into the shelling plants with incoming peanuts; however, the authors note that peanut hulls exit these facilities and are stored near the same point as incoming in-shell peanuts. It is therefore plausible that the source of the insects could be the stored hulls and foreign material associated with the shelling process that accumulated adjacent to the building. As peanuts move by conveyor belts and bucket elevators away from this area, there was a decrease in debris (dust, peanut shells and fungal spores) that accumulated on machinery and floors. Further, the high oil content of nuts inhibits dust emissions farther away from in-shell peanut entry. Therefore, the researchers suggest that sanitation efforts need to be intensely focused on removing debris build up in the early stages of processing to reduce harborage sites for grain feeding insects. In addition to removing refugia, good sanitation improves residual and aerosolized insecticide efficacy [12,14,31].

*Lasioderma serricorne* was the most common insect species captured in number and frequency in dome traps across locations. While there were orders of magnitude higher captures in dome traps compared to flight traps, this is explainable by the presence of the pheromone lure in the dome traps. The authors did not anticipate *L. serricorne* to be the dominant species and would have included an *L. serricorne* lure in the flight traps presumably resulting in greater captures. These observations suggest that *L. serricorne* is an excellent indicator of general insect activity and infestation throughout the facility. Managers may want to focus on this species using only *L. serricorne* pheromones to alleviate having to identify multiple beetle species that are recruited to the Quick-Change^TM^ Ultra-Combi^TM^ lure. *Lasioderma serricorne* is common among food storage and processing facilities due to its unique biology. Although peanut hulls are a poor food source compared to shelled peanuts, *L. serricorne* hosts a yeast-like symbiont, *Symbiotaphrina kochii* Jurzitza (Taphrinaceae), in specialized tissues within the fore and midgut [32]. This symbiont produces essential nutrients (co-enzymes) and aids in detoxification thereby allowing the beetle to thrive on low quality food substances [33,34].

*Cadra cautella* was the only insect that occurred in similar numbers inside and outside the warehouse. This observation suggests that this species is likely immigrating into the shelling plants from outside sources, such as peanut warehouses or other surrounding habitats. Previous studies conducted on populations of *P. interpunctella* and *C. cautella* conclude that these species could be emigrating from product warehouses nearby, as few were captured at great distances from product storage sites [35,36]. This could be contributing to trends found in indoor flight traps throughout this study, indicating moth population emigration from peanut warehouses, on shelling plant grounds, and the movement into processing facilities.

Practitioners of integrated pest management focus their efforts on preventative measures including population monitoring, sanitation and client education in addition to insecticide applications [7]. A non-significant regression of counts over distance for fungal feeding species suggests relative spatial uniformity within processing plants. Given that counts of these species were low, this observation may suggest that an obvious source is not present and therefore these species may be immigrating into the facilities [11,37]. The authors hypothesize that management for fungus feeders may be accomplished through simple building repairs or preventative measures such as closing doors, sealing entry routes, and applying residual insecticides [11]. Conversely, significant regressions of counts over distance for *L. serricorne* and *T. castaneum* suggest that these populations are likely developing in the processing area. Since previous research clearly shows that improved sanitation could decrease population development, the authors hypothesize that spending additional energy focused in the processing area would be prudent. Studies testing these hypotheses are necessary before commercial recommendations are extended.

## 5. Conclusions

Peanut shelling plants harbored many different insect species including fungus and grain/legume feeders. Warmer temperatures always led to increased insect captures in both trap types across facilities. Results suggest that shelling plant managers should focus insect management efforts towards minimizing harborage locations near where in-shell peanuts enter the shelling plants. While *L. serricorne* and *Tribolium* spp. were representative of general insect activity and infestation potential, managers could focus specifically on *L. serricorne* captures in pheromone-baited traps to avoid having to sort through multiple species that may or may not be important.

## Figures and Tables

**Table 1:** Insect species, total number captured and frequency (% of 48 intervals) of capture for all insects in dome traps and flight traps located inside and outside peanut shelling plant 1.

Family and Species	Inside Dome Traps		Inside Flight Traps		Outside Flight Traps	
	Sum Captured	Frequency (%)	Sum Captured	Frequency (%)	Sum Captured	Frequency (%)
Anthicidae						
*Anthicus* sp.	29	33				
Anthocoridae	22	27				
Blattidae					34	4
Carabidae	17	21			25	28
Cicadellidae					31	32
Curculionidae						
*Sitophilus zeamais*	10	21				
Dermaptera	147	63				
Dermestidae					29	8
*Trogoderma variabile*	126	54	44	29	1304	78
Elateridae					8	16
Formicidae	55	38			8	8
Gelechiidae						
*Sitotroga cerealella*	5	8	55	15	5	6
Ichneumonoidea	16	15	3	4	22	14
Laemophloeidae						
*Cryptolestes* spp.	34	38	5	8	9	1
Mycetophagidae						
*Typhaea stercorea*	355	90	4	8	14	22
Nitidulidae						
*Carpophilus* spp.	262	67	4	6		
Ptinidae						
*Lasioderma serricorne*	13,424	98	296	52	14	12
Pyralidae						
*Cadra cautella*	10	19	505	71	31	32
*Ephestia elutella*			1	2		
*Ephestia kuehniella*			14	4	7	12
*Plodia interpunctella*	8	15	625	73	57	32
Scarabaeidae					111	18
Scolytidae			1	2	60	30
Silvanidae						
*Ahasverus advena*	53	52	1	2	6	8
*Oryzaephilus mercator*	26	33				
Staphylinidae	72	60	1	2	241	70
Tenebrionidae						
*Tribolium confusum*	5	8				
*Tribolium castaneum*	829	96				

**Table 2:** Insect species, total number captured and frequency (% of 48 intervals) of capture for all insects in dome traps and flight traps located inside and outside peanut shelling plant 2.

	Inside Dome Traps		Inside Flight Traps		Outside Flight Traps	
Family and Species	Sum Captured	Frequency (%)	Sum Captured	Frequency (%)	Sum Captured	Frequency (%)
Anthocoridae	147	24				
Dermaptera	32	43				
Dermestidae	28	41	1	1	54	24
*Trogoderma variabile*	53	43	37	37	11,020	80
Elateridae					16	24
Formicidae	15	15			275	33
Ichneumonoidea	9	17				
Laemophloeidae						
*Cryptolestes* spp.	946	87	29	30	175	50
Mycetophagidae						
*Typhaea stercorea*	83	65				
Nitidulidae						
*Carpophilus* spp.	1439	85				
Ptinidae						
*Lasioderma serricorne*	41,375	100	308	57		
Pyralidae						
*Cadra cautella*	46	48	1451	89	1467	91
*Ephestia elutella*			12	7	34	21
*Ephestia kuehniella*			167			
*Plodia interpunctella*	8	9		43	627	61
Silvanidae						
*Ahasverus advena*	166	67				
*Oryzaephilus mercator*	132	72				
*Oryzaephilus*						
*surinamensis*	15	9				
Staphylinidae	13	20			142	59
Tenebrionidae	28	17				
*Tribolium confusum*	820	93				
*Tribolium castaneum*	1793	100	15	17		
*Palorus subdepressus*	19	11				
Trogossitidae	11	15				
